# Zerumbone ameliorates behavioral impairments and neuropathology in transgenic APP/PS1 mice by suppressing MAPK signaling

**DOI:** 10.1186/s12974-020-01744-1

**Published:** 2020-02-17

**Authors:** Lei Li, Xiang-Hui Wu, Xiao-Jing Zhao, Lu Xu, Cai-Long Pan, Zhi-Yuan Zhang

**Affiliations:** 1 Department of Pathology, Nanjing Medical University, Longmian Avenue 101, Nanjing, 211166 China; 2 The Key Laboratory of Antibody Technique of Ministry of Health, Nanjing Medical University, Nanjing, 211166 China; 3 Department of Neurology, Sir Run Run Hospital, Nanjing Medical University, Nanjing, 211166 China; 4 Division of Immunopathology of the Nervous System, Institute of Pathology and Neuropathology, University of Tuebingen, Calwer Street 3, D-72076 Tuebingen, Germany

**Keywords:** Alzheimer’s disease, APP/PS1 transgenic mouse, Zerumbone, Cerebral amyloidosis, Neuroinflammation, MAPK signaling pathway

## Abstract

**Background:**

Alzheimer’s disease (AD) is a major clinical problem, but there is a distinct lack of effective therapeutic drugs for this disease. We investigated the potential therapeutic effects of zerumbone, a subtropical ginger sesquiterpene, in transgenic APP/PS1 mice, rodent models of AD which exhibit cerebral amyloidosis and neuroinflammation.

**Methods:**

The N9 microglial cell line and primary microglial cells were cultured to investigate the effects of zerumbone on microglia. APP/PS1 mice were treated with zerumbone, and non-cognitive and cognitive behavioral impairments were assessed and compared between the treatment and control groups. The animals were then sacrificed, and tissues were collected for further analysis. The potential therapeutic mechanism of zerumbone and the signaling pathways involved were also investigated by RT-PCR, western blot, nitric oxide detection, enzyme-linked immunosorbent assay, immunohistochemistry, immunofluorescence, and flow cytometry analysis.

**Results:**

Zerumbone suppressed the expression of pro-inflammatory cytokines and induced a switch in microglial phenotype from the classic inflammatory phenotype to the alternative anti-inflammatory phenotype by inhibiting the mitogen-activated protein kinase (MAPK)/nuclear factor-kappa B signaling pathway in vitro. After a treatment period of 20 days, zerumbone significantly ameliorated deficits in both non-cognitive and cognitive behaviors in transgenic APP/PS1 mice. Zerumbone significantly reduced β-amyloid deposition and attenuated pro-inflammatory microglial activation in the cortex and hippocampus. Interestingly, zerumbone significantly increased the proportion of anti-inflammatory microglia among all activated microglia, potentially contributing to reduced β-amyloid deposition by enhancing phagocytosis. Meanwhile, zerumbone also reduced the expression of key molecules of the MAPK pathway, such as p38 and extracellular signal-regulated kinase.

**Conclusions:**

Overall, zerumbone effectively ameliorated behavioral impairments, attenuated neuroinflammation, and reduced β-amyloid deposition in transgenic APP/PS1 mice. Zerumbone exhibited substantial anti-inflammatory activity in microglial cells and induced a phenotypic switch in microglia from the pro-inflammatory phenotype to the anti-inflammatory phenotype by inhibiting the MAPK signaling pathway, which may play an important role in its neuroprotective effects. Our results suggest that zerumbone is a potential therapeutic agent for human neuroinflammatory and neurodegenerative diseases, in particular AD.

## Background

Alzheimer’s disease (AD) is the major cause of dementia and the most common form of neurodegeneration. The clinical hallmarks of this disease include impairments in learning and memory, in complex attention, executive function, language, visuospatial function, praxis, gnosis, and behavior and/or social comportment [1]. Furthermore, the development of senile plaques composed of extracellular accumulated β-amyloid (Aβ) peptides [2] is one of the most distinct neuropathological features of AD. It is hypothesized that Aβ peptides are toxic and contribute to memory loss, behavioral impairments, and further neurodegenerative pathology in AD [3, 4]. In addition to Aβ aggregation, neuroinflammation also plays a pivotal role in the pathophysiology of AD [5]. Neuroinflammation is characterized by microglial and astroglial activation, and the release of numerous inflammatory mediators, particularly around amyloid plaques [6, 7]. In AD, excessive production and inefficient clearance of Aβ lead to Aβ aggregation and trigger a pro-inflammatory response, which contributes to cell death and neural dysfunction [8]. This chronic neuroinflammation promotes Aβ liberation during amyloid precursor protein (APP) processing [9]. All of the aforementioned neuropathological changes establish a deleterious self-perpetuating vicious cycle which promotes the pathogenesis of AD [10]. Therefore, controlling neuroinflammation and reducing Aβ accumulation in the AD brain are thought to be promising approaches for the treatment of AD [10].

Microglia are the resident phagocytes of the central nervous system (CNS), and their phagocytic capacity can be regulated to enhance the clearance of Aβ [11, 12]. This suggests that reversing microglial phenotype from the disease state to the cognitively normal phenotype or specifically activating microglia could be effective therapeutic approaches for AD [13, 14]. In a recent study, deferoxamine treatment switched the microglial phenotype from the classic to the alternative phenotype, thereby decreasing Aβ_42_ deposition and ameliorating cognitive impairments in APP/PS1 mice [15]. It is also reported that gamma oscillations induced a distinct microglial phenotype, and thereby promoted the microglial phagocytosis of Aβ in 5 × FAD mice [16]. The classically activated (M1) pro-inflammatory and alternatively activated (M2) anti-inflammatory phenotypes are two different microglial polarization states [17]. Alternatively activated microglia have been reported to possess a significantly increased phagocytic capacity for Aβ compared to classically activated microglia [18]. Classically activated pro-inflammatory microglia are characterized by an increased production of pro-inflammatory mediators, including tumor necrosis factor-α (TNF-α), interleukin-1β (IL-1β), and interleukin-6 (IL-6) [19]. These cells exacerbate neuronal damage and impede cellular repair in several neurodegenerative diseases [20]. Conversely, alternatively activated anti-inflammatory microglia are known to upregulate anti-inflammatory mediators, such as arginase-1 (Arg-1), CD206, and interleukin-10 (IL-10) [21]. These cells exert neuroprotection and promote neuronal recovery and remodeling [22]. Therefore, understanding how to control neuroinflammation and enhance microglial phagocytosis is key for the development of novel AD treatments.

The mitogen-activated protein kinase (MAPK) signaling pathway mediates fundamental cellular and biological processes related to immune responses. This pathway consists of three major classes of MAPKs, namely, extracellular signal-regulated kinase (ERK), c-Jun N-terminal kinase (JNK), and p38 [23, 24]. Impaired and uncontrolled regulation of the MAPK pathway has been implicated in a number of immunological disorders including microglia dysfunction and neuroinflammation [25], suggesting its involvement in CNS disorders. Previous studies have revealed that the MAPK signaling pathway plays an important role in AD pathogenesis activation of this pathway was observed in AD brains and pathologically-similar brains [26, 27]. ERK and p38 were observed to be closely associated with AD pathology, especially with Aβ accumulation in microglia that responded strongly to β-amyloid [25]. Inhibition of MAPK signaling reverses memory impairments in mouse models of AD [28, 29] and is reported to promote microglial anti-inflammatory (M2) polarization and thereby inhibit neuroinflammation [30, 31]. Taken together, improving microglial function via the regulation of MAPK signaling may be a promising strategy for the treatment of AD.

Zerumbone (2,6,9,9-tetramethyl-2E,6E,10E-cycloundeca-2,6,10-trien-1-one) is a sesquiterpenoid compound extracted from the rhizomes of *Zingiber zerumbet Smith*, a wild ginger which primarily grows in Southeast Asia [32–34]. In traditional Asian medicine, the rhizome of this plant is considered to have anti-rheumatic, anti-spasmodic, carminative, and analgesic effects [35]. Indeed, recent studies have indicated that zerumbone has several pharmacological effects, namely, anti-cancer, chemo-preventive, and anti-inflammatory effects [33]. Zerumbone has been shown to reduce the expression of inflammatory cytokines and molecules in different inflammatory-induced cell cultures [36, 37]. For example, zerumbone suppressed nuclear factor-kappa B (NF-κB), inducible nitric oxide synthase (iNOS), and TNF-α expression, reduced malondialdehyde accumulation, and increased glutathione and glutathione reductase levels in mice with UVB-induced photokeratitis [38]. In a mouse model of neuropathic pain, zerumbone exhibited anti-allodynic and anti-hyperalgesic activities via the suppression of IL-1β, IL-6, and TNF-α [39]. Notably, the inhibition of these pro-inflammatory markers by zerumbone is accompanied by the attenuation of the MAPK signaling pathway in lipopolysaccharide (LPS)-induced macrophages [36]. Moreover, several other sesquiterpenoids have been suggested to possess anti-neuroinflammatory activity or control Aβ production in the brain, identifying them as potential therapeutic agents for AD [40, 41]. However, the effect of zerumbone on neurodegenerative disorders, especially AD, is currently unknown.

Hence, a transgenic mouse model (C57BL/6 J genetic background) co-expressing mutated human amyloid precursor protein (APP) and presenilin 1-21 (PS1-21) (APP/PS1-21 mice) was used in our in vivo study. Although this transgenic mouse model resembles more directly the rare familial variants of AD (FAD) cases accounting for early-onset autosomal dominant forms than the over 95% onsets of sporadic AD, current AD research mainly depends on FAD, especially for the most of the animal models. FAD fully represents amyloid theory which is the best known and accepted theory describing molecular mechanisms of AD. And most importantly, transgenic models could only be designed from involved genes like APP and PS genes in these familial forms. These mice exhibit aggressive AD pathology, specifically neuroinflammation and impairments in behavioral/cognitive functions [42, 43]. We used a murine N9 microglial cell line and primary microglia from mouse brains to study the therapeutic potential of zerumbone for AD in vitro, and to explore the potential effects of zerumbone on MAPK signaling. Our results may facilitate the development of novel therapeutic approaches for the treatment of AD.

## Methods

### Animals

Male APP/PS1-21 mice on a C57BL/6 J background were obtained from Prof. M. Jucker (University of Tuebingen, Germany). Heterozygous male APP/PS1-21 mice were bred with wild-type C57BL/6 J females (Charles River Germany, Sulzfeld, Germany). Offspring were tail-snipped and genotyped using PCR with primers specific for the APP-sequence (forward, “GAATTCCGACATGACTCAGG”; reverse, “GTTCTGCTGCATCTTGGACA”). Animals were housed under a 12-h light/dark cycle with free access to food and water. All experiments and protocols were licensed and approved by the local government in Germany, according to The German Animal Welfare Act (TierSchG) of 2006; or by Nanjing Medical University Animal Care and Use Committee in accordance with the regulations of the ethics committee of the International Association for the Study of Pain and the Guide for the Care and Use of Laboratory Animals (The Ministry of Science and Technology of China, 2006) in China.

### Reagents

**Fig. 1:**
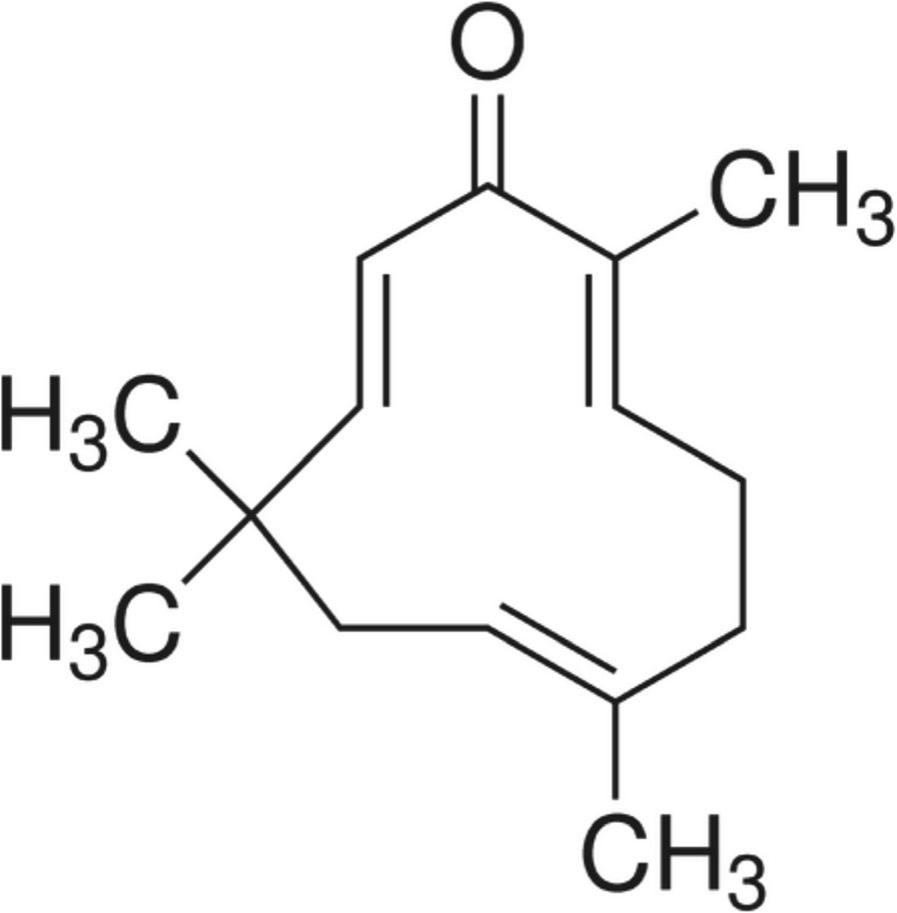
Molecular structure of zerumbone

DMEM/F-12 medium, fetal bovine serum (FBS), penicillin, and streptomycin were purchased from Gibco (Waltham, MA, USA). The Aβ used in these experiments was human Aβ_1–42_ (ChinaPeptides, Shanghai, China). The Aβ powder was dissolved and incubated in DMSO, and was then diluted to a stock solution (500 μM) with phosphate buffered saline (PBS; Boster Biological Technology, Wuhan, China). Before the experiments, we incubated the mixed stock solution at 37 °C for 24 h. Zerumbone (Zer, > 98% of Purity, Fig. 1) and lipopolysaccharide (LPS) were purchased from Sigma-Aldrich (Munich, Germany). Zerumbone was dissolved in DMSO to produce a 20 μM stock solution. Same volume or amount of DMSO and/or PBS was applied as vehicles. For the in vitro experiments, stock solutions of Aβ or zerumbone were further diluted to different working concentrations with culture media. After 6 h of pretreatment of Zer or vehicle, we treated the cells with the 10 μM Aβ_1–42_ for 12 h. The dose of amyloid-beta used in cell culture experiments is based on our previous studies and is consistent with the majority of previous in vitro studies on AD. We tested serial concentrations of amyloid-beta from 1 to 30 μM and 10 μM was an appropriate concentration, which did not harm the cellular activity in our cell culture.

### Cell culture

Primary microglia were isolated from the cortex of newborn (postnatal day 0–2) C57BL/6 J mice by mild trypsinization as previously described [44]. Briefly, the cortex was chopped and digested into a single cell suspension which was incubated in DMEM/F-12 containing 10% FBS and 100 U/ml 1% penicillin/streptomycin. After 15 days of incubation, mixed glial cells were shaken for 2 h at 37 °C. Detached microglia were then harvested for the subsequent experiments. In some experiments, primary microglia were also isolated from brain homogenates as previously described with minor modifications [45]. Briefly, the brains of zerumbone-treated or control mice were minced and finely homogenized in ice-cold Hank’s Balanced Salt Solution (HBSS, pH 7.4). The homogenates were centrifuged twice at 250 g for 5 min. The pellet was re-suspended in 1 ml 70% isotonic Percoll (GE Healthcare, Uppsala, Sweden). A discontinuous Percoll density gradient was layered as follows: 70%, 50%, and 35% isotonic Percoll, and PBS. The gradient was centrifuged at 1200 g for 45 min at 4 °C, and microglia were collected from the 70/50% interphase Percoll layers.

Isolated cells were washed and re-suspended in sterile HBSS, and were then stained with PE-conjugated anti-CD11b and FITC-conjugated anti-CD45 antibodies (1:1000, Serotec, Oxford, UK). The purity of CD11b^+^/CD45^low^ microglia was confirmed to be > 85%. The primary microglia were grown in DMEM medium at 37 °C and 5% CO_2_. All cultures were supplemented with penicillin/streptomycin (100 U/ml) and 10% FBS. Subsequently, 10^5^ cells were seeded onto 12-well cell culture plates and cultured.

### Cell viability assays

Cell viability was evaluated using a Cell Counting Kit-8 (CCK-8; Yeasen, Shanghai, China). Briefly, after each treatment, 10 μl of CCK-8 reagent was added and microglia were incubated for 4 h at 37 °C and 5% CO_2_. The absorbance of the samples was measured at 450 nm using a microplate reader. Cell viability was determined using the following calculation: cell viability (%) = (A (stimulated)–A (blank)/(A (control)–A (blank) × 100%.

### RNA isolation and real-time PCR

To determine the effects of zerumbone on the inflammatory response of microglia in vitro, N9 microglial cells and primary microglial cells were used. As previously mentioned, all cells were cultured in DMEM medium with 100 U/ml penicillin/streptomycin and 10% FBS. The cells were seeded in 12-well plates and were then divided into three groups. Cells in the vehicle group were treated with a solution of Aβ_1–42_. Cells in the Aβ group were treated with 10 μM Aβ_1–42_ for 24 h. Cells in the Zer group were pre-treated with zerumbone (at concentrations of 1, 3, or 10 μg/ml) for 24 h before treatment.

Total RNA was extracted from cells using the RNeasy Mini Kit (QIAGEN, Hilden, Germany) following the manufacturer’s protocol. The QuantiTect Reverse Transcription Kit (QIAGEN) was used to reverse transcribe RNA (1 μg) into cDNA. Real-time PCR analysis was performed using ChamQ SYBR qPCR Master Mix (Vazyme, Nanjing, China) to measure the mRNA expression of IL-1β, IL-6, iNOS, TNF-α, CD206, IL-10, and ARG-1. The amplification efficiency of these primers had previously been established using calibration curves. Each 20 μl qPCR reaction mixture contained 3 μl water, 1 μl forward primer (20 μmol/l), 1 μl reverse primer (20 μmol/l), 10 μl MasterMix, and 50 ng cDNA. PCR amplification was conducted as follows: denaturation at 95 °C for 10 min, 40 cycles of 95 °C for 10 s and 65 °C for 60 s, followed by 1 cycle at 97 °C for 1 s. Finally, a melting step was performed consisting of 10 s at 95 °C, 60 s at 65 °C, and slow heating at a rate of 0.1 °C per second to 97 °C with continuous fluorescence measurement. Quantification was performed using the comparative CQ method (2^−ΔΔCQ^). The expression of each target mRNA was calculated relative to that of β-actin (four samples from each group were analyzed by PCR) [46, 47].

### Nitric oxide detection and enzyme-linked immunosorbent assay

Supernatants from different wells were collected and a standard Griess assay (Sigma-Aldrich) was performed to analyze the production of nitric oxide (NO). Enzyme-linked immunosorbent assay (ELISA) kits for IL-1β (Thermo Scientific, Waltham, MA, USA), TNF-α, IL-10, and prostaglandin E2 (PGE2; BioLegend Inc., San Diego, CA, USA) were used to detect the concentrations of these cytokines in microglial culture supernatants. To further confirm the role of the MAPK pathway in the effects of zerumbone, microglia were pre-treated for 2 h with 10 μM U0126 (an ERK inhibitor), SB202190 (a p38 inhibitor), or BAY 11–7082 (a NF-κB inhibitor), and further cultured with 3 μg/ml zerumbone. ELISAs were then conducted to determine the effects of zerumbone on the release of IL-1β, TNF-α, and IL-10. Protein from half-brains was serially extracted to produce soluble and insoluble protein fractions. Brains were homogenized with a tissue mite homogenizer for 15 s in PBS twice, and then sonicated. Homogenates were cleared by centrifugation at 12,000*g* for 45 min at 4 °C. The resulting supernatant was labeled as the soluble fraction. The pellet was re suspended in 1 ml of guanidine hydrochloride (5 M guanidine hydrochloride in 1 M Tris, pH 8.0) by pipetting followed by rotation overnight at room temperature and labeled as the insoluble fraction. The concentrations of Aβ were also measured by ELISA kit (Wako, Osaka, Japan) at A450 nm. All samples were analyzed four times.

### Flow cytometry analysis

To detect the effects of zerumbone on Aβ phagocytosis, microglia were stimulated with Aβ_1–42_ and incubated with or without zerumbone (3 μg/ml) for 24 h. Each tube of cells was incubated with 1 μl (500 ng/μL) Aβ_1–42_ (HiLyte™ Fluor 488-labeled, Eurogentec, Liege, Belgium) for 1 h at 4 °C. Data were analyzed using FlowJo Software (Version 7.6.1; TreeStar, Ashland, OR, USA).

### Western blot analysis

To identify the signaling pathway involved in the effects of zerumbone, total protein was extracted from primary microglia and the brains of vehicle- and zerumbone-treated APP/PS1 mice using RIPA lysis buffer (50 mM Tris, 150 mM NaCl, 1% TritonX-100, 1% sodium deoxycholate, and 1% SDS). The volumes and contents of all samples were equalized with RIPA lysis buffer, and the samples were electrophoretically separated on 12% SDS-PAGE gels. Following this, the proteins were transferred to PVDF membranes (Millipore, Billerica, MA, USA) using Trans-Blot apparatus (Bio-Rad, Hercules, CA, USA). The membranes were blocked with Tris-buffered saline solution (TBS) containing 5% bovine serum albumin (BSA) for 2 h, and were then incubated at 4 °C overnight with primary antibodies against cyclooxygenase-2 (Cox-2), microsomal prostaglandin E synthase-1 **(**m-PGES-1), ERK, p38 MAPK, NF-κB p65 (1:1000, Abcam, Cambridge, MA, USA), and β-actin (1:500, Sigma, St. Louis, MO, USA). The proteins were visualized using appropriate horseradish peroxidase-conjugated secondary antibodies and an enhanced chemiluminescence reagent. The signals of specific proteins were detected using a Gel Doc imager (Bio-Rad) and were expressed as a fraction of the signal of the control protein [48, 49].

### Mouse treatment and groups

In order to be administered orally, zerumbone was suspended in 1% carboxymethylcellulose (CMC, Blanose®, Hercules-Aqualon, Düsseldorf, Germany) at a concentration of 3.5 mg/ml (zerumbone/CMC solution). Five-month-old mice were divided into two groups. Group 1 comprised seven APP/PS1-21 mice (five males and two females) which were treated for 20 days with zerumbone (25 mg/kg by daily gavage). Group 2 comprised seven sex- and age-matched APP/PS1-21 mice which were administered the same volume (200 μl) of 1% CMC dissolved in water.

### Design and evaluation of nest construction assay

A nest construction assay [50] was modified to identify deficits in the affiliative/social behavior of APP/PS1 mice and potential changes following treatment.

For at least 24 h, mice were individually housed in clean plastic cages with wood chip bedding (approximately 1 cm deep) lining the floor. Identification cards were coded to render the experimenter blind to the sex, age, and genotype of the mice. Two hours prior to the onset of the dark phase of the light/dark cycle, a 20 × 20 cm piece of paper towel torn into approximately 5 × 5 cm square pieces were placed in each cage. Mice were tested in balanced groups of mixed genotypes to reduce variability in housing conditions. The next morning (approximately 16 h later), the cages were inspected for nest construction. Pictures were taken prior to evaluation for documentation. Paper towel nest construction was scored using a 5-point system: 1 = no biting or tears on the paper, 2-3 = moderate biting and/or tears on the paper but no coherent nest (not grouped into a corner of the cage), and 4-5 = the vast majority of paper torn into pieces and grouped into a corner of the cage [50, 51].

### Social interaction: resident-intruder assay

The social interaction assay was performed according to previous studies [51, 52] with minor modifications. The resident-intruder assay was video-recorded to evaluate all distinct behaviors of vehicle-treated and zerumbone-treated WT or AD mice (residents) in the presence and absence of an intruder mouse, and to analyze the movement of the mice to assess their overall activity level and overt neurobiological differences. In this assay, the observer was blinded to treatment allocation. A mouse was placed in a clean plastic cage identical to its home cage (325 mm × 210 mm × 185 mm) for 15 min to establish it as the “resident” mouse. An age-, weight-, and sex-matched untreated naive mouse (the “intruder mouse”) was then introduced for a second 15-min period.

The numbers of independent (15 min without intruder and 15 min with intruder) and interactive behavioral events (15 min with intruder) were counted for the resident mouse. Independent behavioral events included sniffing the environment, rearing alongside the cage, rearing independently, digging, circling clockwise, circling counter-clockwise, allogrooming, freezing, and scratching. Interactive behavioral events included sniffing the other mouse, following, grooming, rearing at the other mouse, sitting or lying next to the other mouse, backing or running away from the other mouse, biting, boxing or wrestling, mounting, pinning, and tail-rattling. The recorded videos were analyzed, and the numbers of events were counted by three independent observers blinded to treatment allocation.

To calculate the total distance traveled and quantify all identifiable distinct behaviors, both 15-min sessions were videotaped at a frame rate of 15 Hz. This sampling rate ensured that fast movements of the mice were sufficiently captured and allowed for a fine-grained analysis of the trajectory of these movements, but maintained manageable file sizes. A region of interest in the captured video (500 × 310 pixels) was saved directly to a computer for later analysis.

Following the acquisition of the video, the position of the mouse was localized in each frame. Tracking was performed using Java-based software (Oracle, Redwood City, CA, USA) [53]. To determine the location of the mouse in each frame, the pixel of maximum intensity was identified, and a subset image around this pixel was extracted. The center of intensity of the subset image was calculated and used to determine the object’s *X* and *Y* locations. The total distance traveled per transgenic mouse was then calculated using mouse behavior analysis software developed in our lab.

### Novel object recognition test

In the training phase of the novel object recognition test, each mouse was placed into the experimental arena with two identical green objects (5 × 5 × 5 cm) and allowed to explore for 10 min. Twenty-four hours later, the novel object preference test (10 min) was conducted. The mouse was placed in the arena and presented with two objects in the same position as before, one familiar object and one novel red plastic object (3.5 × 4 × 6 cm). The length of time that the animal spent exploring the novel object was recorded. The recognition index (RI) was determined using the following calculation: recognition index = the length of time spent exploring the novel object/the total length of time spent exploring both objects × 100.

### Morris water maze test

The Morris water maze (MWM) test was conducted in a circular tank (120 cm diameter, 60 cm height). The depth of the water was 0.3 m and the temperature of the water was 23–25 °C. A platform (7.5 cm diameter) was placed in one quadrant of the pool, 13 cm from the edge. After 1 day of habitation, training was initiated, and each mouse underwent three 1-min trials per day. After 4 days of training, the probe test was then conducted. The escape latency of each mouse, defined as the time taken to find the hidden platform, was recorded and analyzed.

### Immunohistochemistry and image analysis

Zerumbone- and vehicle-treated mice were sacrificed after 20 days of treatment. The mice were deeply anesthetized with ether and perfused intracardially with ice-cold 4% paraformaldehyde in PBS. The brains were quickly removed and post-fixed in 4% paraformaldehyde overnight at 4 °C. The post-fixed brains were cut into two hemispheres. Both hemispheres were embedded in paraffin, serially sectioned (3-μm thickness), and mounted on silane-covered slides. These sections were then stained via immunohistochemistry as described previously [54]. The following antibodies were used: anti-Aβ (1:100, Abcam) for Aβ deposition, anti-ionized calcium-binding adaptor molecule-1 (Iba-1; 1:200, Wako, Neuss, Germany) for activated microglia, and anti-CD206 (1:100, Biorbyt, Cambridge, UK) for M2 microglia. The rabbit polyclonal anti-Aβ antibody (ab2539) was generated against the synthetic peptide DAEFRHDSGYEVHH conjugated to Keyhole Limpet Hemocyanin (KLH), corresponding to amino acids 1–14 of human Aβ.

CD206, widely known as the mannose receptor, is expressed by many types of cell, including macrophages and various epithelial cells. CD206 is expressed by microglia with an anti-inflammatory phenotype, but not by those with a pro-inflammatory phenotype, and is therefore used as a marker of anti-inflammatory microglia [55]. A double-staining experiment with anti-Iba-1 and anti-CD206 antibodies was performed. After the brain tissue sections were immune-labeled with Iba-1 as previously described, they were once more irradiated and incubated with the primary anti-CD206 antibody for 2 h at 20 °C. Immunostaining was developed with Fast Blue BB salt chromogen-substrate solution but counterstaining with hemalum was not performed.

After immunostaining, the hemisphere sections were examined using a light microscope (Nikon Coolscope; Nikon, Tokyo, Japan). Aβ and Iba-1 immunostaining were evaluated in the hemisphere sections, particularly in the cortex and the hippocampus. All sections were randomly numbered and analyzed independently by two observers, who were blinded to the treatment and time points. The numbers of Aβ plaques and Iba-1-positive cells in the cortex and hippocampus were counted under a microscope at × 50 magnification. Small Aβ plaques with a dense core and larger plaques with a dense core and a large halo of diffuse amyloid were counted. Small areas or spots of Aβ staining, smaller than a cellular nucleus (approximately 10 μm of diameter), and slightly stained diffuse amyloid without a dense core were classified as unclear deposition and were not counted. Furthermore, images of the hemisphere sections were captured using a Nikon Coolscope (Nikon) with fixed parameters. The cortex and the hippocampus were outlined on the images and analyzed using MetaMorph Offline 7.1 software (Molecular Devices, Toronto, Canada). The percentage areas of Aβ, Iba-1, CD206, and CD206 + Iba-1 in the regions of interest were determined using color threshold segmentation. All parameters were fixed for all images of a specific stain. Results are presented as the arithmetic mean of plaque/cell counts or percentage areas of immunoreactivity (IR) in an area of interest in the cross-sections and the standard error of the mean (SEM). Additionally, the ratios of CD206 immunoreactivity to Iba-1 IR and the ratios of double staining IR to Iba-1 or Aβ IR were calculated and presented in bar graphs.

### Immunofluorescence and confocal analysis

For the NF-κB activation assay, vehicle-, Aβ-, and zerumbone-treated N9 cells were plated in confocal cell culture dishes. The cells were then fixed with ice-cold methanol, permeabilized with 0.25% Triton X-100/TBST, and blocked with 1% BSA in TBST for 1 h. The cells were incubated with an anti-p65/RelA antibody (1:100, Abcam) for 2 h at room temperature and Alexa Fluor 647-conjugated secondary antibodies (1:1000, Cell Signaling Technology, Beverly, MA, USA) at room temperature for 1 h. To identify synaptic loss, the brains of AD mice with or without Zerumbone treatment were fixed with paraformaldehyde, paraffin-embedded, and serially sectioned into 3 μm thickness. These sections were incubated with primary antibody against synapse (1:300, Abcam) overnight at 4 °C and Alexa Fluor 488-conjugated secondary antibody (1:500, Abcam) for 1 h at room temperature. Finally, the nuclei were stained with 4′, 6-diamidino-2-phenyl-indole (DAPI; SouthernBiotech, Birmingham, Ala, USA). Confocal microscopy was carried out using a Zeiss Axiovert system (LSM510; Carl Zeiss, Jena, Germany).

To detect Aβ in brain tissue, anesthetized animals were perfused transcardially with 4% paraformaldehyde. The brains were immediately removed, post-fixed in the same paraformaldehyde solution, and transferred to 30% sucrose solution at 4 °C. The specimens were then embedded in Tissue-Tek® O.C.T compound and frozen at − 80 °C. Specimens were cut into 25-μm-thick sections by a freezing microtome (CM1860; Leica, Wetzlar, Germany) and incubated overnight at 4 °C with antibodies against Aβ (1:200, Abcam, Iba-1, or glial fibrillary acidic protein (GFAP; 1:500, Novus Biologicals, Abingdon, UK). After washing, the sections were incubated with Alexa Fluor-conjugated secondary antibodies for 1 h at room temperature. Cell nuclei were stained with DAPI. Images of each section were captured using a fluorescence microscope (Leica) by investigators blinded to the experimental groups.

To analyze microglial morphology, images were captured using a confocal microscope (LSM800, Zeiss) with × 20 magnification. ImageJ (Loci, Madison, WI, USA) was used to analyze the shape of two-dimensional somatic projections in confocal images of Iba-1-immunostained microglia based on maximum length (L) and projection area (A) [56].

### Statistical analysis

Differences in plaque/cell counts, areas of staining, and nest construction scores between the vehicle- and zerumbone-treated groups were analyzed by the Mann-Whitney *U* test or the Kruskal-Wallis test if more than two groups are compared, using GraphPad Prism 5.0 software (San Diego, CA, USA). The data are represented as median and interquartile ranges for non-parametric statistics. For all statistical analyses, a *p* value < 0.05 was considered statistically significant.

## Results

### Zerumbone reduces inflammation and promotes the phenotypic conversion of microglia in vitro

**Fig. 2:**
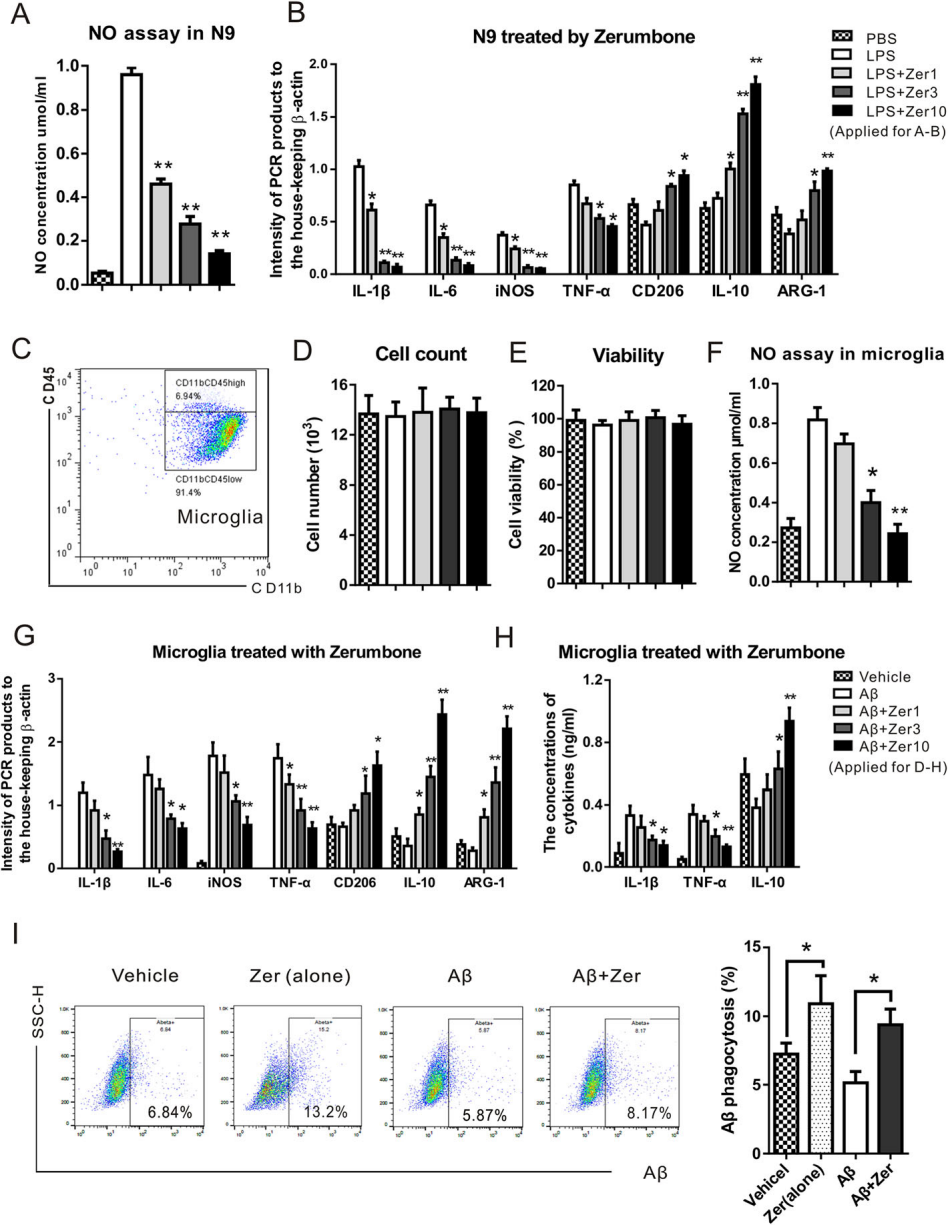
Zerumbone reduces inflammation and promotes the phenotypic conversion of microglia in vitro. The murine N9 microglial cell line and primary microglia isolated from newborn mice were stimulated with lipopolysaccharide (1 μg/ml) or β-amyloid (Aβ; 10 μM), and incubated with or without zerumbone (1, 3, or 10 μg/ml) for 24 h. **a** Bar graph showing the nitric oxide (NO) concentration in N9 cells. Zerumbone treatment significantly reduced NO production. **b** Bar graph showing the expression levels of indicated genes including interleukin-1β (IL-1β), interleukin-6 (IL-6), inducible nitric oxide synthase (iNOS), tumor necrosis factor-α (TNF-α), CD206, interleukin-10 (IL-10), and arginase-1 (ARG-1) in N9 cells. Zerumbone treatment significantly decreased the mRNA levels of IL-1β, IL-6, iNOS, and TNF-α, and increased the mRNA levels of CD206, IL-10, and ARG-1 in a dose-dependent manner. **c** The purity of primary microglia was identified using flow cytometry, and CD11b^+^/CD45^low^ cells were confirmed as microglia. **d**, **e** Cell counting kit-8 (CCK8) experiments showed that neither Aβ nor different doses of zerumbone affected cell number or viability. **f** Bar graph showing the NO concentration in primary microglia cells. Zerumbone treatment significantly reduced NO production. **g** Bar graph showing the expression levels of indicated genes including IL-1β, IL-6, iNOS, TNF-α, CD206, IL-10, and ARG-1 in primary microglia. Zerumbone treatment significantly decreased the mRNA levels of IL-1β, IL-6, iNOS, and TNF-α, and increased the mRNA levels of CD206, IL-10, and ARG-1 in a dose-dependent manner. **h** Bar graph showing the concentrations of cytokines (IL-1β, TNF-α, and IL-10) in the cell culture supernatants of primary microglia. Zerumbone treatment significantly decreased the levels of IL-1β and TNF-α, and increased the levels of IL-10. **i** Fluorescence-activated cell sorting (FACS) results and a bar graph showing that zerumbone increased the phagocytosis of Aβ in microglia cells. Data are presented as median and interquartile ranges (*n* = 5). *p < 0.05, **p < 0.01

The in vitro anti-inflammatory effects of zerumbone were analyzed using the murine N9 microglial cell line and primary microglia. LPS was used to induce inflammatory microglial activation. LPS-stimulated N9 microglial cells exhibited increased NO production (Fig. 2a) and mRNA expression of IL-1β, IL-6, iNOS, and TNF-α (Fig. 2b), which are characteristic markers of the pro-inflammatory phenotype and thus indicate inflammatory microglial activation. Following LPS stimulation, zerumbone treatment significantly reduced NO production and dose-dependently decreased the mRNA levels of IL-1β, IL-6, iNOS, and TNF-α, suggesting that zerumbone has an anti-inflammatory effect on microglia. The mRNA levels of CD206, IL-10, and ARG-1, markers of the anti-inflammatory phenotype were significantly increased by zerumbone administration, indicating that zerumbone promotes a switch in microglial phenotype from the classic pro-inflammatory phenotype to the anti-inflammatory phenotype. Furthermore, primary microglia were isolated from mouse brains to identify the effects of zerumbone in vitro. First, the purity of primary microglia was identified using flow cytometry, and CD11b^+^/CD45^low^ microglia accounted for over 85% of the cells assayed (Fig. 2c). As shown in Fig. 2d, e, compared to the control cells, neither the numbers nor the viabilities of Aβ- or zerumbone-treated microglia showed significant differences. The Aβ-treated primary microglia exhibited increased NO production and mRNA expression of inflammatory factors including IL-1β, IL-6, iNOS, and TNF-α. Zerumbone also dose-dependently increased the mRNA levels of anti-inflammatory factors such as CD206, IL-10, and ARG-1 (Fig. 2f, g). The expression levels of IL-1β, TNF-α, and IL-10 in primary microglia were detected using ELISA (Fig. 2h). Zerumbone significantly reduced the production of IL-1β and TNF-α. Additionally, zerumbone-treated microglia exhibited significantly increased IL-10 production compared to Aβ treated microglia. Therefore, zerumbone exhibited anti-inflammatory activity and induced a switch in microglial polarization towards the anti-inflammatory phenotype, suggesting that it may have further applications in neuroinflammation-related diseases. The effects of zerumbone on microglial Aβ phagocytosis were investigated using flow cytometry. Zerumbone-treated microglia exhibited increased Aβ phagocytosis (Fig. 2i).

### Zerumbone attenuates Aβ-induced MAPK/NF-κB activation in microglia

**Fig. 3:**
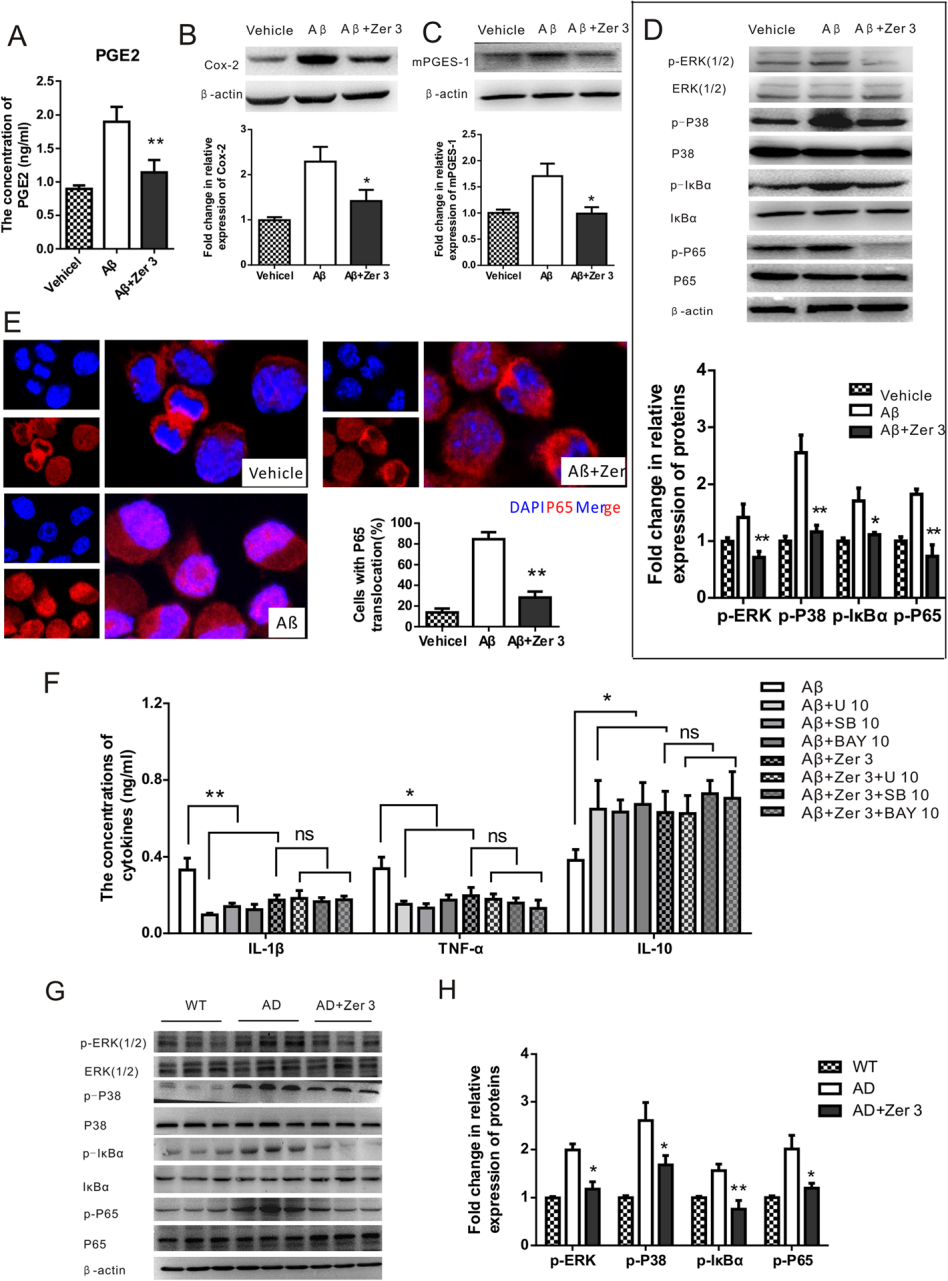
Zerumbone attenuates mitogen-activated protein kinase activation in microglial cells. **a** Bar graph showing the concentration of prostaglandin E2 (PGE2) in N9 cells. The production of PGE2 was significantly reduced by zerumbone. **b** Representative western blots and a bar graph showing the protein expression of cyclooxygenase-2 (Cox-2) in N9 cells. The production of Cox-2 was significantly reduced by zerumbone. **c** Representative western blots and a bar graph showing the protein expression of microsomal prostaglandin E synthase-1 (mPGES-1) in N9 cells. The production of mPGES-1 was significantly reduced by zerumbone. **d** Representative western blots and a bar graph showing the protein expression levels of p-extracellular signal-regulated kinase (p-ERK), ERK, p-p38, p38, p-IκBα, IκBα, p-p65, and p65 in N9 cells. Zerumbone treatment significantly decreased the levels of p-ERK, p-p38, p-IκBα, and p-p65. **e** Zerumbone inhibited the translocation of nuclear factor-kappa B (NF-κB) from the cytosol to the nucleus in N9 cells treated with Aβ for 24 h. **f** The concentrations of cytokines (interleukin-1β (IL-1β), tumor necrosis factor-α (TNF-α), and interleukin-10 (IL-10)) in the cell culture supernatants of N9 microglia. Zerumbone, U0126, SB202190, and BAY 11-7082 blocked the Aβ induced up-regulation of IL-1β and TNF-α production. The Aβ-induced decrease in IL-10 production was also reversed by zerumbone, U0126, SB202190, and BAY 11-7082. Data are presented as median and interquartile ranges (*n* = 4). Zer1, 3, 10 = zerumbone concentrations of 1, 3, or 10 μg/ml. U, U0126 (an ERK inhibitor); SB, SB202190 (a p38 inhibitor); BAY, BAY 11–7082 (an NF-κB inhibitor). **g**, **h** Primary microglia were isolated from the cortex of vehicle- and zerumbone-treated APP/PS1 mice. The microglia isolated from zerumbone-treated mice exhibited decreased protein levels of p-ERK1/2, p-p38, p-IκBα, and p-p65 compared to vehicle-treated mice. Data are presented as median and interquartile ranges (*n* = 3). *p < 0.05, **p < 0.01

Recent studies have indicated that zerumbone is a potent suppressor of the p38 MAPK/NF-κB signaling pathway. Hence, we investigated the effects of zerumbone on the expression of PGE2, Cox-2, and mPGES-1, which are all related to p38 MAPK, and four important mediators of the MAPK/NF-κB pathway in Aβ treated microglia. Treatment with zerumbone suppressed the expression of PGE2, Cox-2, mPGES-1, and the phosphorylated forms of ERK1/2, p38 MAPK, inhibitor of kappa B alpha (IκBα), and p65 NF-κB compared to treatment with Aβ (Fig. 3a–d). As shown in Fig. 3e, Aβ induced the translocation of p65 NF-κB into the nucleus, and zerumbone treatment significantly reduced this translocation.

To provide further evidence that the anti-inflammatory activity of zerumbone is related to inhibition of the ERK/p38 MAPK/NF-κB signaling pathway, we further inspected the effect of ERK/p38 MAPK/NF-κB-specific inhibitors on the concentrations of cytokines in the cell culture supernatants of microglia. As shown in Fig. 3f, U0126 (an ERK inhibitor), SB202190 (a p38 inhibitor), and BAY 11-7082 (an NF-κB inhibitor) blocked the Aβ-stimulated upregulation of IL-1β and TNF-α production. Similarly, the Aβ-induced decrease in IL-10 production was also reversed by U0126, SB202190, and BAY 11-7082. Interestingly, when microglia were treated with zerumbone, none of the three inhibitors altered the secretion of inflammatory factors. These results suggested that zerumbone induced a switch in microglial polarization towards the anti-inflammatory phenotype by down-regulating the Aβ stimulated activation of the ERK/p38 MAPK/NF-κB signaling pathway in microglia.

### Zerumbone treatment attenuates the activation of MAPKs in microglia from APP/PS1 transgenic AD model mice

We further investigated the activation of the ERK/P38MAPK/NF-κB signaling pathway in microglia of vehicle- and zerumbone-treated APP/PS1 transgenic AD model mice. The microglia isolated from zerumbone-treated mice exhibited decreased p-ERK1/2 expression compared to those isolated from vehicle-treated mice (Fig. 3g, h). Western blot analysis revealed that the expression of p-p38 was significantly decreased in microglia of zerumbone-treated APP/PS1 mice. Moreover, zerumbone significantly suppressed the phosphorylation of p65 NF-κB in microglia of APP/PS1 mice. These results indicate that zerumbone attenuates the activation of the MAPK/NF-κB signaling pathway in microglia of APP/PS1 transgenic AD model mice.

### Zerumbone treatment alleviates behavioral impairments in APP/PS1 transgenic AD model mice

**Fig. 4:**
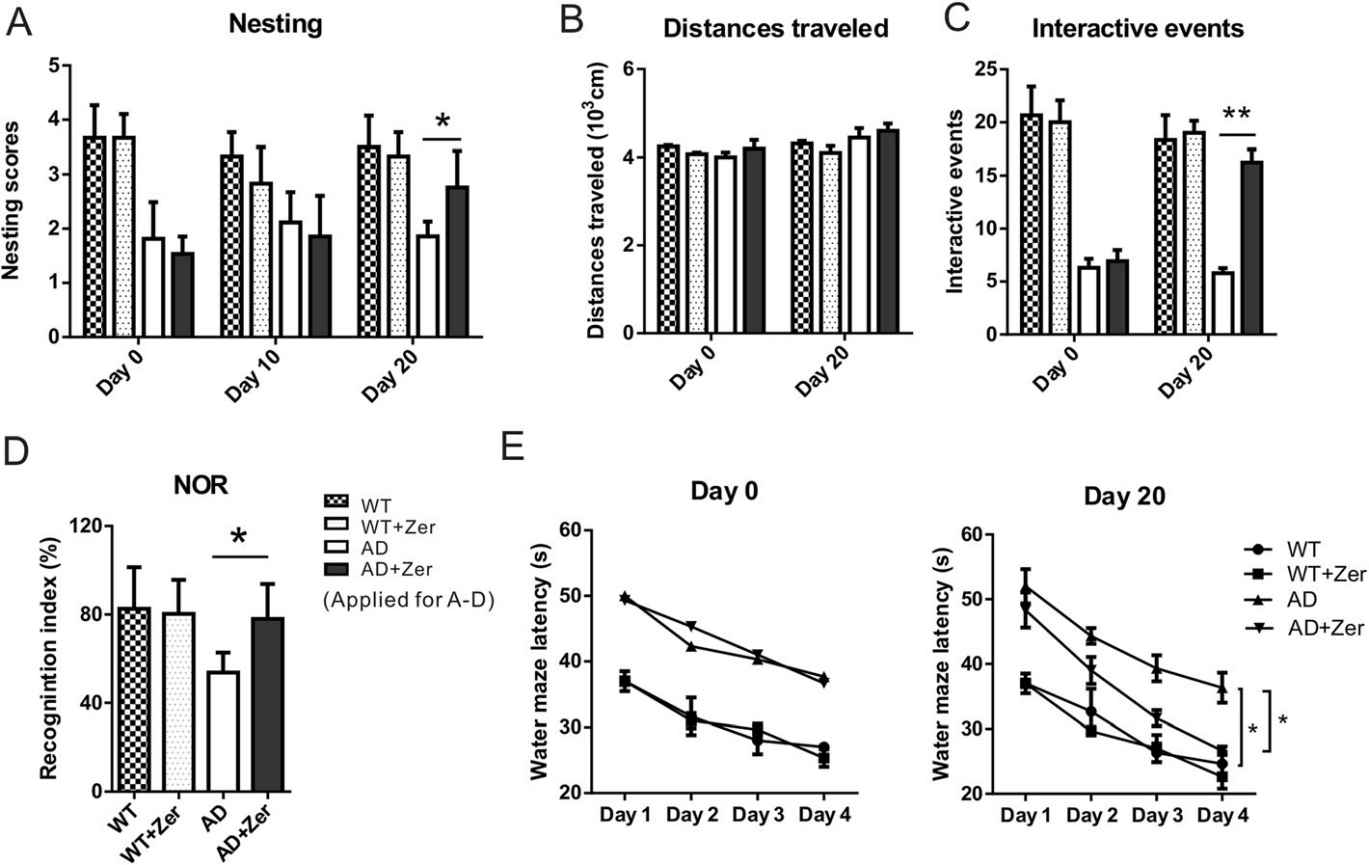
Zerumbone alleviates behavioral impairments in APP/PS1 mice. APP/PS1 mice were treated for 20 days with zerumbone (or carboxymethylcellulose (CMC), vehicle) by gavage. **a** A nest construction assay was conducted involving a 3-point scale. The nest-building scores at day 0 and day 10 were not significantly different between the vehicle- and zerumbone-treated APP/PS1 mice. At day 20, zerumbone-treated mice exhibited significantly higher nest-building scores compared to vehicle-treated APP/PS1 mice. **b**, **c** A resident-intruder assay was conducted, and the distances traveled and numbers of interactive events were analyzed. Bar graph showing the distances traveled by vehicle- and zerumbone-treated APP/PS1 mice before and after zerumbone administration for 20 days. The vehicle- and zerumbone-treated mice did not exhibit significant differences in distances traveled at any time point (**b**). **c** Bar graph showing the number of interactive events of vehicle- and zerumbone-treated APP/PS1 mice before and after 20 days of treatment. At day 20, zerumbone-treated mice exhibited a significantly higher frequency of interactive behavior. **d** In the novel object recognition test, the length of time that the mouse spent exploring the novel object was recorded and the recognition index was calculated. The bar graph shows the recognition index of vehicle- or zerumbone-treated wild-type (WT) and APP/PS1 mice. At day 20, zerumbone significantly increased the recognition index of APP/PS1 mice. **e** Zerumbone ameliorated cognitive deficits in APP/PS1 mice in the hidden platform test of the Morris water maze test. All mice showed shorter escape latency on the fourth day, and APP/PS1 mice showed impaired cognitive function compared to WT mice. Zerumbone-treated APP/PS1 mice showed significant improvements in cognitive function during the 4 days of training compared to the vehicle-treated APP/PS1 mice. Data are presented as median and interquartile ranges (*n* = 7). *p < 0.05, **p < 0.01

For all the following behavioral tests, we have included two groups of wild-type (WT) mice of comparable age to the AD transgenic mice: one was control and the other one was treated with Zerumbone. Behavioral tests showed that WT mice had no deficits in cognitive or non-cognitive behaviors, and Zerumbone treatment did not induce any change in these WT mice (Fig. 4).

To assess the influence of zerumbone treatment on nesting behavior, a nest construction assay was conducted involving a 3-point scoring system. WT mice had no deficits in nesting ability at all the time points, and Zerumbone treatment did not induce any change in these mice. In one of our previous studies, we showed that the nesting ability of transgenic APP/PS1 mice was impaired. At day 0 (before zerumbone treatment), no significant difference was observed between the vehicle- and zerumbone-treated APP/PS1 mice with respect to nest construction score. Similarly, after 10 days of treatment (day 10), no significant difference in nest construction score was noted between these two groups. At day 20, however, a significant difference was observed in the nest construction scores of vehicle- and zerumbone-treated APP/PS1 mice (Fig. 4a). The vehicle-treated APP/PS1 mice investigated and slightly bit the paper towels, but did not destroy them. The towels were found distributed throughout the cage and were not grouped into a corner. In contrast, the zerumbone-treated APP/PS1 mice bit the paper towels, tore them into small pieces, and grouped them into a corner of the cage.

In the social interaction assay, the movements of the mice were recorded by two cameras: a vertically placed camera which was used to calculate the distances traveled by the mice, and a camera placed at the side of the cage which was used to count the numbers of individual and interactive behaviors. Both cameras were adjusted to a suitable height to ensure that all animal movements were captured. The distances traveled by the animals, according to X/Y coordinates, were transformed into actual distances (pixel to centimeter). The distances traveled by the vehicle- and zerumbone-treated mice were not significantly different before or after treatment (Fig. 4b), indicating that the motor function of mice in both groups was normal and was not affected by the treatment. Two unfamiliar mice placed in the same cage will often display high levels of interaction. The videos were analyzed to determine the frequency of carefully defined behavioral events. WT mice had no deficits in social interaction during the entire experiment, and Zerumbone treatment did not induce any change in these mice. Prior to treatment, the frequency of interactive behaviors did not significantly differ between the vehicle- and zerumbone-treated APP/PS1 mice. Following 20 days of treatment, resident zerumbone-treated APP/PS1 mice showed a significantly higher frequency of interactive behaviors compared to vehicle-treated APP/PS1 mice (Fig. 4c).

In the novel object recognition test, WT mice explored the novel object for a longer time period and exhibited a RI of approximately 81%, indicating their ability to remember the familiar object. In contrast, APP/PS1 mice exhibited a significantly lower RI of 48%, demonstrating that the cognitive function of these animals was impaired. When given daily for 20 days, zerumbone significantly increased the RI of APP/PS1 mice to approximately 78%, showing that zerumbone ameliorated cognitive impairments in these mice (Fig. 4d).

The MWM test is one of the most sensitive tests for AD-like deficits as it examines the function of the hippocampus, the region of the brain most affected in AD. To assess hippocampal spatial memory deficits, the mice were placed into a water tank, and the length of time it took them to locate the hidden platform was recorded. The performance of mice with or without zerumbone treatment was evaluated. There were no significant differences between vehicle- and zerumbone-treated APP/PS1 mice, indicating that these animals had similar motor and visual capabilities. The escape latencies of each mouse were compared to assess the learning and memory ability of the mice. The vehicle-treated APP/PS1 mice exhibited a significantly increased escape latency compared to WT mice, demonstrating that the learning ability of these animals was impaired. Zerumbone-treated APP/PS1 mice performed significantly better and exhibited a significantly reduced escape latency compared to the controls (Fig. 4e).

### Zerumbone treatment reduces microglial activation and the number of amyloid plaques in APP/PS1 transgenic AD model mice

**Fig. 5:**
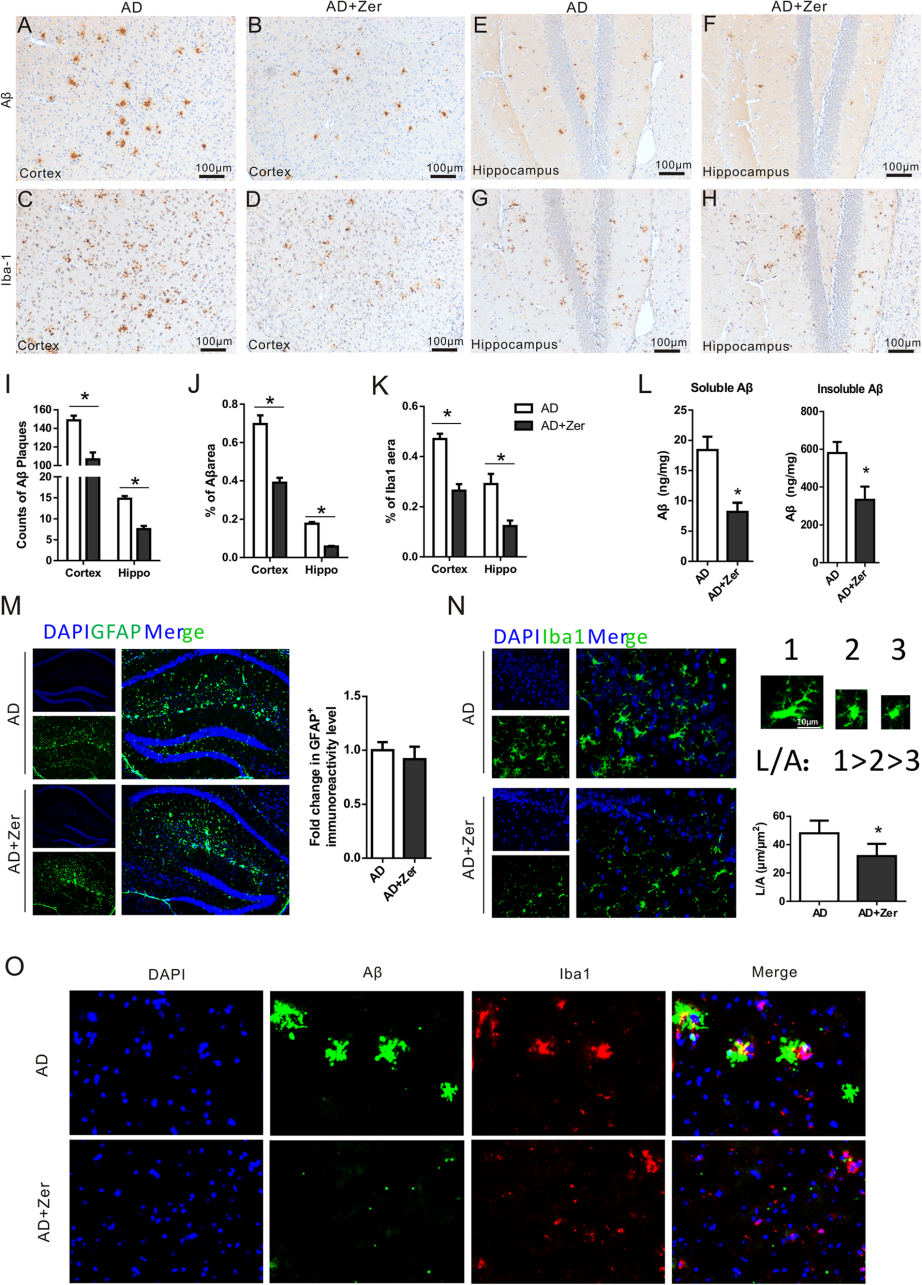
Zerumbone decreases Aβ deposition and microglial activation in APP/PS1 mice. Five-month-old APP/PS1–21 mice, five males and two females, were treated for 20 days with zerumbone (25 mg/kg by daily gavage). **a**–**d** Representative photomicrographs of β-amyloid (Aβ) immunohistochemical staining and ionized calcium-binding adaptor molecule 1 (Iba-1) immunohistochemical staining in the cortex of vehicle- and zerumbone-treated APP/PS1 mice. Zerumbone reduced Aβ deposition and microglial activation in the cortex of APP/PS1 mice. **e**–**h** Representative photomicrographs of Aβ immunohistochemical staining and Iba-1 immunohistochemical staining in the hippocampus of vehicle- and zerumbone-treated APP/PS1 mice. Zerumbone reduced Aβ deposition and microglial activation in the hippocampus of APP/PS1 mice. Scale bar in (**a**–**h**) = 100 mm. **i** Quantification of the numbers of Aβ plaques in the cortex and hippocampus of APP/PS1 mice. **j** Quantification of the percentage areas of the Aβ staining in the cortex and hippocampus of APP/PS1 mice. **k** Quantification of the percentage areas of the Iba-1 staining in the cortex and hippocampus of APP/PS1 mice. **l** Soluble and insoluble Aβ protein levels were measured in half-brain homogenates by ELISA. AD mice treated with Zerumbone decreased the concentration of Aβ in brains significantly. **m** Representative photomicrographs of glial fibrillary acidic protein (GFAP) immunofluorescence in the hippocampus of vehicle- and zerumbone-treated APP/PS1 mice. Statistical analysis showed no significant differences between the groups. **n** Shape analyses of two-dimensional somatic projections based on maximum length (L) and projection area (A) in confocal images of Iba-1-immunostained microglia using ImageJ. The somatic shape index (L:A ratio) increases in rod-shaped somata. The L, A, and L:A ratio of microglial somata were measured. **o** Representative photomicrographs of Aβ and Iba-1 double immunofluorescence in the cortex of vehicle- and zerumbone-treated APP/PS1 mice. Zerumbone treatment increased the phagocytosis of Aβ by Iba-1^+^ microglia (*n* = 7). Data are presented as median and interquartile ranges. *p < 0.05

In APP/PS1 AD model mice, amyloid plaques were spread throughout the entire cortex, while no Aβ staining could be seen in the brains of WT mice (data not shown). Some plaques were large and had a dense core and large area of diffused amyloid, but small plaques with a dense core were also observed (Fig. 5a). Plaque density was generally lower in the hippocampus than in the cortex (Fig. 5e). Compared with vehicle-treated mice, there were more Aβ plaques with fewer branches and smaller size in zerumbone-treated mice (Fig. 5b, f, and o). Zerumbone treatment attenuated neuropathological histological changes compared to vehicle-treated APP/PS1 mice. Zerumbone treatment significantly reduced the numbers of plaques in the cortex and the hippocampus (Fig. 5i), and substantially decreased the areas of Aβ IR in the cortex and the hippocampus (Fig. 5j). Moreover, soluble Aβ is particularly neurotoxic and is responsible for major brain dysfunction. Soluble and insoluble Aβ in brains were further investigated using ELISA tests and both of them were decreased in APP/PS1 mice treated with Zerumbone in comparison to those treated with vehicle (Fig. 5l).

In comparison to Aβ staining, amoeboid Iba-1-positive microglia were mostly found clustered around amyloid plaques in both the cortex and hippocampus (Fig. 5c, g). Following zerumbone treatment, the number of Iba-1-positive cells was reduced, especially in the cortex, and these cells were less clustered around amyloid plaques (Fig. 5d, h). Further analysis showed that the percentage areas of Iba-1 IR in the cortex and hippocampus were significantly reduced (Fig. 5k). These results indicate that zerumbone reduced the general activation of Iba-1-positive microglia. In WT mouse brains there was only very little positive Iba-1 staining, which indicated inactivation of the majority of microglia in these brains. No significant changes in microglia number or morphology were observed in WT mice that received Zerumbone treatment, compared to the control WT mice (data not shown). In addition to microglia, we also assessed astrocytic GFAP staining by immunofluorescence analysis. As shown in Fig. 5m, there were no significant differences between vehicle- and zerumbone-treated APP/PS1 mice with respect to astrocytic GFAP staining.

We also assessed the distinct activation of Iba-1^+^ microglia by evaluating microglial shape using image analysis of two-dimensional somatic projections. Shape parameters were calculated based on the maximum length (L) and projection area (A) of individual microglia. Activated microglia exhibit a decreased somatic shape index (L/A). As shown in Fig. 5n, the L:A ratio of microglia from zerumbone-treated mice was smaller than that of microglia from vehicle-treated mice. These results suggest that zerumbone treatment activated microglia in APP/PS1 mice. The co-labeling of Aβ and Iba-1 also showed that zerumbone treatment increased the phagocytosis of Aβ by Iba-1^+^ microglia, decreasing the size of Aβ plaques (Fig. 5o). Taken together, these results demonstrate that although the total number of generally activated microglia was reduced by zerumbone, Aβ phagocytosis and the distinct activation of microglia were promoted by zerumbone treatment.

### Zerumbone treatment increases the proportion of anti-inflammatory microglia in APP/PS1 transgenic AD model mice

**Fig. 6:**
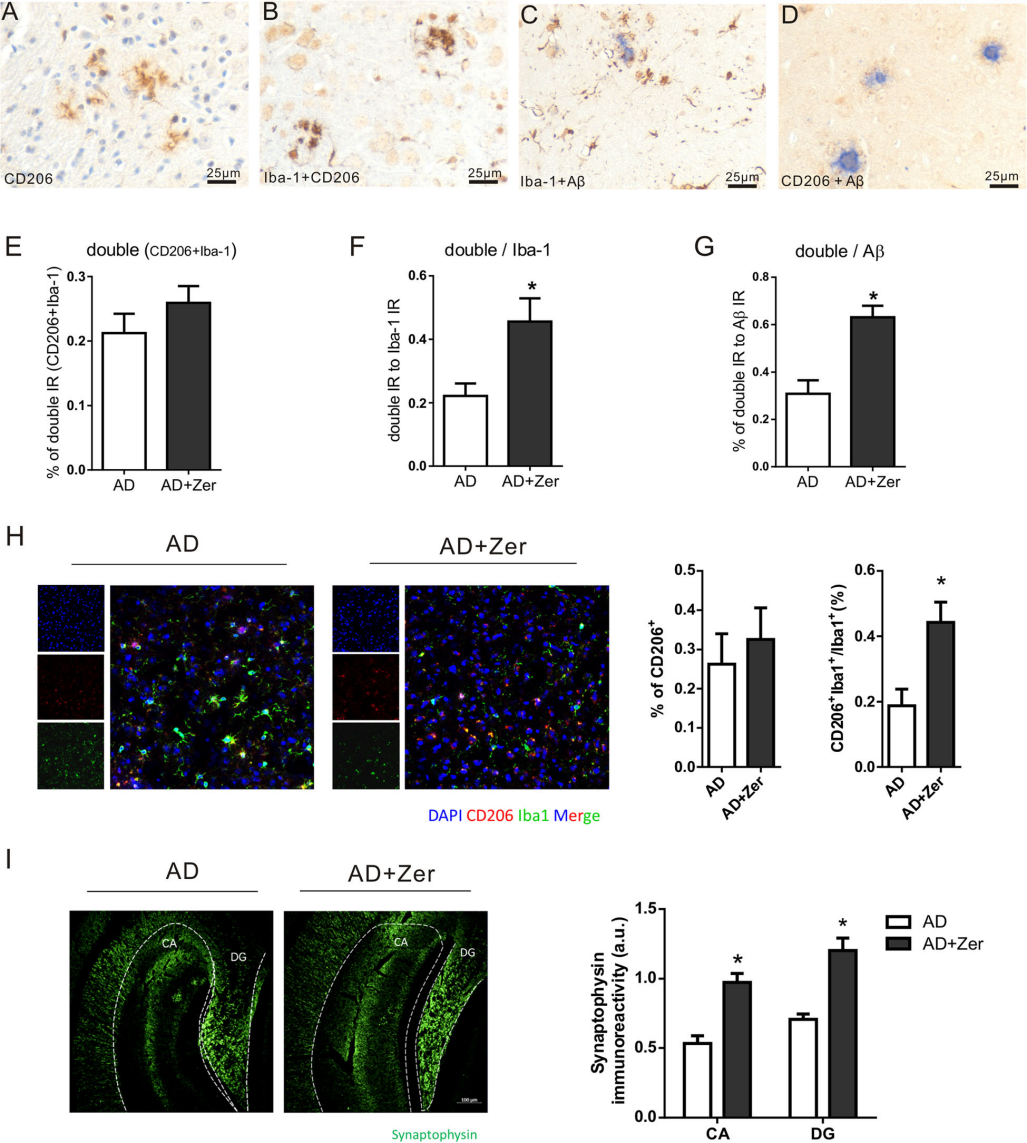
Zerumbone increases the expression of CD206 (an anti-inflammatory marker) in the cortex of APP/PS1 mice. Five-month-old APP/PS1-21 mice, five males and two females, were treated for 20 days with zerumbone (25 mg/kg by daily gavage). **a** Representative photomicrographs of CD206 immunohistochemical staining in the cortex of APP/PS1 mice. **b** Representative photomicrographs of CD206 and ionized calcium-binding adaptor molecule 1 (Iba-1) double immunohistochemical staining in the cortex of APP/PS1 mice. **c** Representative photomicrographs of Iba-1 and β-amyloid (Aβ) double immunohistochemical staining in the cortex of APP/PS1 mice. **d** Representative photomicrographs of CD206 and Aβ double immunohistochemical staining in the cortex of APP/PS1 mice. **e** Quantification of the percentage of CD206 and Iba-1 in the cortex of vehicle- and zerumbone-treated APP/PS1 mice. The areas of CD206 IR and Iba-1^+^CD206 double IR in the cortex were slightly increased by zerumbone treatment. **f** Quantification of the ratio of the area of Iba-1 + CD206 double IR to the area of Iba-1 IR in the cortex of vehicle- and zerumbone-treated APP/PS1 mice. The percentage of Iba-1 + CD206 double staining area to Iba-1 IR was significantly increased by zerumbone treatment. **g** Quantification of the ratio of the area of Iba-1 + CD206 double IR to the area of Aβ IR in the cortex of vehicle and zerumbone-treated APP/PS1 mice. The ratio of the area of Iba-1 + CD206 double IR to the area of Aβ IR was significantly increased by zerumbone treatment. **h** Representative photomicrographs of CD206 and Iba-1 immunofluorescence double staining in the cortex of APP/PS1 mice. The percentage areas of CD206 and the ratio of the area of Iba-1 + CD206 double IR to the area of Iba-1 IR were determined (*n* = 7). *p < 0.05. **i** Representative immunofluorescence images, and quantitative analysis of synaptophysin in the hippocampal CA3 and in the DG, in the brains of Zerumbone treated AD mice and vehicle-treated AD mice (Scale bars, 100 μm) (*n* = 5)

It has been shown that CD206 is mainly expressed by alternatively activated anti-inflammatory (M2) macrophages/microglia, and can therefore be used as a marker of these particular cell types. Although CD206 is a marker of anti-inflammatory microglia, it may also be expressed by non-monocytes. Therefore, in addition to CD206 immunostaining (Fig. 6a), Iba-1 + CD206 (Fig. 6b), Iba-1 + Aβ (Fig. 6c), and CD206 + Aβ (Fig. 6d) double immunostaining were also conducted (IHC). Zerumbone treatment only slightly increased the numbers of CD206^+^ microglia-like cells in cortex or hippocampus (data not shown). The areas of CD206 IR and Iba-1 + CD206 double IR in the cortex were also only slightly increased following zerumbone treatment (Fig. 6e). Given that the area of Aβ IR and the total number of microglia were reduced following zerumbone treatment, ratios of CD206, and Iba-1 + CD206 IR areas compared to Iba-1 IR and Aβ IR were further analyzed. The ratios of the area of CD206 IR to the areas of Iba-1 IR or Aβ IR, the ratios of the area of Iba-1 + CD206 double staining IR to the areas of Iba-1 IR or Aβ IR were all significantly increased (Fig. 6f, g). Our results were further confirmed by immunofluorescence staining and analysis, as shown in Fig. 6h. All these results indicate that zerumbone treatment increased the proportion of anti-inflammatory microglia.

In addition, synaptic loss was determined using the presynaptic marker synaptophysin on these brain sections. A significantly higher immunoreactivity specific to synaptophysin was observed in Zerumbone treated APP/PS1 mice compared to vehicle-treated APP/PS1 mice (Fig. 6i). This result indicates an ameliorated synaptic loss following zerumbone treatment.

## Discussion

In this study, we demonstrated that zerumbone suppressed neuroinflammation and induced a switch in microglial phenotype from the classic inflammatory phenotype to the alternative anti-inflammatory phenotype by inhibiting the MAPK/NF-κB signaling pathway in vitro. In a mouse model of AD, zerumbone significantly ameliorated deficits in both non-cognitive and cognitive behaviors. It also significantly reduced β-amyloid deposition, attenuated pro-inflammatory microglial activation, and increased the proportion of anti-inflammatory microglia among all activated microglia in the cortex and hippocampus of APP/PS1 mice. Meanwhile, the expression of key molecules of the MAPK signaling pathway, for example, p38 and extracellular signal-regulated kinase, was also downregulated in microglia isolated from the brains of zerumbone-treated APP/PS1 mice.

Increasing evidence has shown that the MAPK signaling pathway plays a crucial role in the pathophysiology of AD; activation of this pathway has been observed in the postmortem brains of AD patients and animal models of AD [26, 27, 57]. MAPK inactivation in the prefrontal cortex was shown to relieve memory deficits in AD model mice [28, 29]. The activation of ERK and p38 MAPK is increased in Aβ-exposed microglia and mouse models of AD with more advanced amyloid pathology [25]. Decreased ERK and p38 MAPK activity reduced Aβ neurotoxicity and reversed memory impairments in mouse models of AD [28, 29, 58, 59]. Moreover, p38 MAPK has been found to play an essential role in the regulation of pro-inflammatory signaling networks and in the biosynthesis of cytokines, including TNF-α and IL-1, in immune cells [60]. p38 MAPK is also highly expressed in brain regions that are crucial for learning and memory in patients with AD and epileptic seizures, and is likely involved in higher brain functions [61]. In addition, a core downstream signaling molecule of the MAPK pathway, NF-κB, a major inflammatory signaling molecule and transcription factor, is activated in the brains of AD patients [62, 63], and is thought to play a key role in AD development and cognitive impairments [64, 65]. Inhibition of ERK and p38 MAPK has been reported to suppress Aβ-induced NF-κB transactivation [66, 67], and NF-κB has also been identified as a target for early intervention in AD [65]. Therefore, inhibition of ERK/p38 MAPK may be a promising therapeutic strategy for the treatment of AD [28, 29]. Many studies have been conducted into the development and design of novel p38αMAPK inhibitors and several have presented significant therapeutic effects in animal models of AD [68, 69]. Inhibiting both ERK and p38 MAPK will undoubtedly have a more significant therapeutic effect on AD. Recent studies have focused on the suppression of activated glial cell-mediated neuroinflammation via indirect MAPK inhibitors such as natural terpenoids [70, 71]. The subtropical ginger sesquiterpene zerumbone has exhibited notable anti-inflammatory effects by suppressing the MAPK signaling pathway and NF-κB activation in different cell types [72, 73]. In accordance with previous studies, zerumbone inhibited the ERK/p38 MAPK signaling pathway and thereby exhibited anti-neuroinflammatory activity in cultured microglial cells in this study. This prompted us to further investigate its potential therapeutic effects in an animal model of AD.

Notably, zerumbone ameliorated behavioral deficits in APP/PS1 mice and significantly decreased Aβ deposition and neuroinflammation in the brains of these mice. Patients with AD typically exhibit cognitive and non-cognitive impairments. Toxic Aβ peptides, APP peptides, and inflammatory responses have been shown to be directly associated with impairments in cognitive function and non-cognitive behaviors [74]. Cognitive impairments in animal models, such as memory and learning deficits, are typically evaluated using the novel object recognition task and the MWM test [75]. Affiliative nesting and interactive social behaviors are considered rodent analogs of the non-cognitive behaviors that are characteristically compromised in human AD [76]. Impairments in these two non-cognitive behaviors have been reported in transgenic APP/PS1 mice [77]. The hippocampus, prefrontal cortex, and other brain regions are thought to be responsible for cognitive function and nesting ability [50, 78], while the frontal cortex is believed to be responsible for social memory and social interaction [79, 80]. Therefore, the cognitive function, nesting ability, and social behavior of APP/PS1 mice may also be impaired by pathological changes in these brain regions, namely, toxic Aβ accumulation and neuroinflammation. Given that zerumbone treatment attenuated Aβ accumulation and reduced neuroinflammation in the cortex and hippocampus after a relatively short period, these neuropathological improvements may have contributed to the improved memory and learning ability, nesting ability, and social interactive behavior of the APP/PS1 mice (Fig. 4). Moreover, many previous studies have shown that Aβ and neuroinflammation interact with each other, which aggravates the progress of synaptic loss, that is directly responsible for many brain dysfunctions, especially cognitive decline [81, 82]. Our results showed ameliorated synaptic loss in the hippocampus following the zerumbone treatment (Fig. 6i). We consider that this improvement might be due to reduced toxic Aβ and neuroinflammation. In addition, behavioral tests and pathological assays showed that Zerumbone has no effects on WT mice, regarding cognitive/non-cognitive behavioral changes, neuroinflammation or gliosis. These results demonstrated the therapeutic effect of zerumbone in this animal model of AD, and suggest that it may have beneficial effects in AD patients.

In addition to the suppression of neuroinflammation, our in vitro results suggested that the therapeutic effects of zerumbone may also be mediated via the switching of microglial phenotype. Previous studies have not directly specified the association of microglial phenotype with MAPK inactivation in AD pathology. The present study demonstrated that ERK/p38 MAPK signaling was affected by zerumbone treatment, with significantly decreased levels of p-p38 and p-ERK found in microglia isolated from zerumbone-treated APP/PS1 mice (Fig. 3g). Importantly, changes in microglial phenotype were closely associated with the neuroprotective effect of zerumbone. Microglial activation and the expression of inflammatory molecules are directly involved in neurodegenerative pathology and in the development of neuroinflammation [83]. A previous study reported a direct correlation between inflammatory status and amyloid load in the cell culture supernatant of organotypic brain slice cultures from a similar transgenic AD mouse model using multi-plex cytokine array analysis [84]. After we observed an ameliorated inflammatory milieu by zerumbone in our microglial culture, histological assays of brain tissue from APP/PS1 mice showed that zerumbone significantly attenuated microglial inflammatory activation in both the cortex and hippocampus (Fig. 4). This indicates that zerumbone controls neuroinflammation and may also ameliorate other pathological changes, since attenuated neuroinflammation is known to contribute to reduced amyloid plaque accumulation [85]. Interestingly, we further observed that zerumbone switched microglial phenotype from the inflammatory phenotype to the anti-inflammatory phenotype in vitro (Fig. 2b, g and h). Furthermore, although the total number of activated microglia reduced, the proportion of anti-inflammatory microglia increased in the brains of zerumbone-treated APP/PS1 mice (Fig. 6). Additionally, microglial Aβ phagocytosis was increased since Aβ deposition around activated microglia was significantly reduced (Fig. 5). These results suggest that zerumbone may play a therapeutic role beyond its anti-neuroinflammatory potential, since alternatively activated anti-inflammatory microglia have been reported to possess a significantly increased capacity for Aβ phagocytosis compared to classically activated pro-inflammatory microglia [18, 86], and were associated with decreased Aβ deposition in a previous study of APP/PS1 mice [87]. Furthermore, our in vitro results suggest that the zerumbone-induced switching of microglial phenotype towards the anti-inflammatory phenotype was mediated by down-regulation of the ERK/p38 MAPK signaling pathway in microglia.

p38 MAPK is reported to be significantly involved in microglial activation and neuroinflammation [88]. p38α KO mice exhibit an increased tendency towards alternatively activated anti-inflammatory (M2) microglial polarization [30], and suppressing p38 MAPK activation in glial cells alleviated neurotoxicity in a mouse model of AD [70, 89]. Recently, it has been found that inhibition of other MAPK signaling molecules, namely, JNK and ERK, also promotes alternative microglial polarization and thereby inhibits neuroinflammation [31]. These results suggest that promoting anti-inflammatory microglial polarization by inhibiting MAPK signaling could be a promising treatment strategy for AD. Similarly, inhibition of the NF-κB pathway by certain non-steroidal anti-inflammatory drugs reduces the levels of pro-inflammatory cytokines and increases the levels of anti-inflammatory cytokines in microglia. But clinical trials testing NSAIDS in AD showed no beneficial effects in humans [90]. This results in the recruitment of distinctly activated Aβ phagocytic microglia and the amelioration of cognitive deficits in AD animal models [91], consistent with the previously established role of distinct microglial activation in AD [83].

Our mouse model exhibits very aggressive AD-like pathology, from 3 to 7 months of ages the development of Aβ deposits presents an exponential growth pattern, the pathological changes at 5 months of age are already well developed [92]. The current study thereby mainly explores early and mid-term drug interventions for AD. Similarly, early intervention is also recommended for the treatment of AD in clinical practice [93]. As aging is a major risk factor for AD, research on aged mice is also important and would be conducted in future work. In recent years, increasing evidence has demonstrated that zerumbone has neuroprotective effects in different cells and experimental animals, but the molecular targets and potential mechanisms of action of zerumbone remain to be fully elucidated. Although we demonstrated that zerumbone ameliorated behavioral impairments and neuropathological changes in transgenic APP/PS1 mice by suppressing MAPK signaling, the specific binding site of zerumbone remains unknown. The molecular mechanism of zerumbone’s therapeutic effects could be better clarified and proved with more pathway studies and more control groups, such as ERK / p38 MAPK signaling pathway knockdown/knockout and overexpression groups. There is no doubt that this will be the focus of our future studies. In this study, we selected the dose of 25 mg/kg orally in mice based on previous animal studies with zerumbone [94, 95]. According to the guide for dose conversion between mice and human, the daily human oral dose would be 2.75 mg/kg, which can be reasonable [96]. Pharmacokinetics of zerumbone in WT and transgenic mice will also be determined in the future to provide direct supportive data for potential therapeutic applications in humans.

## Conclusion

**Fig. 7:**
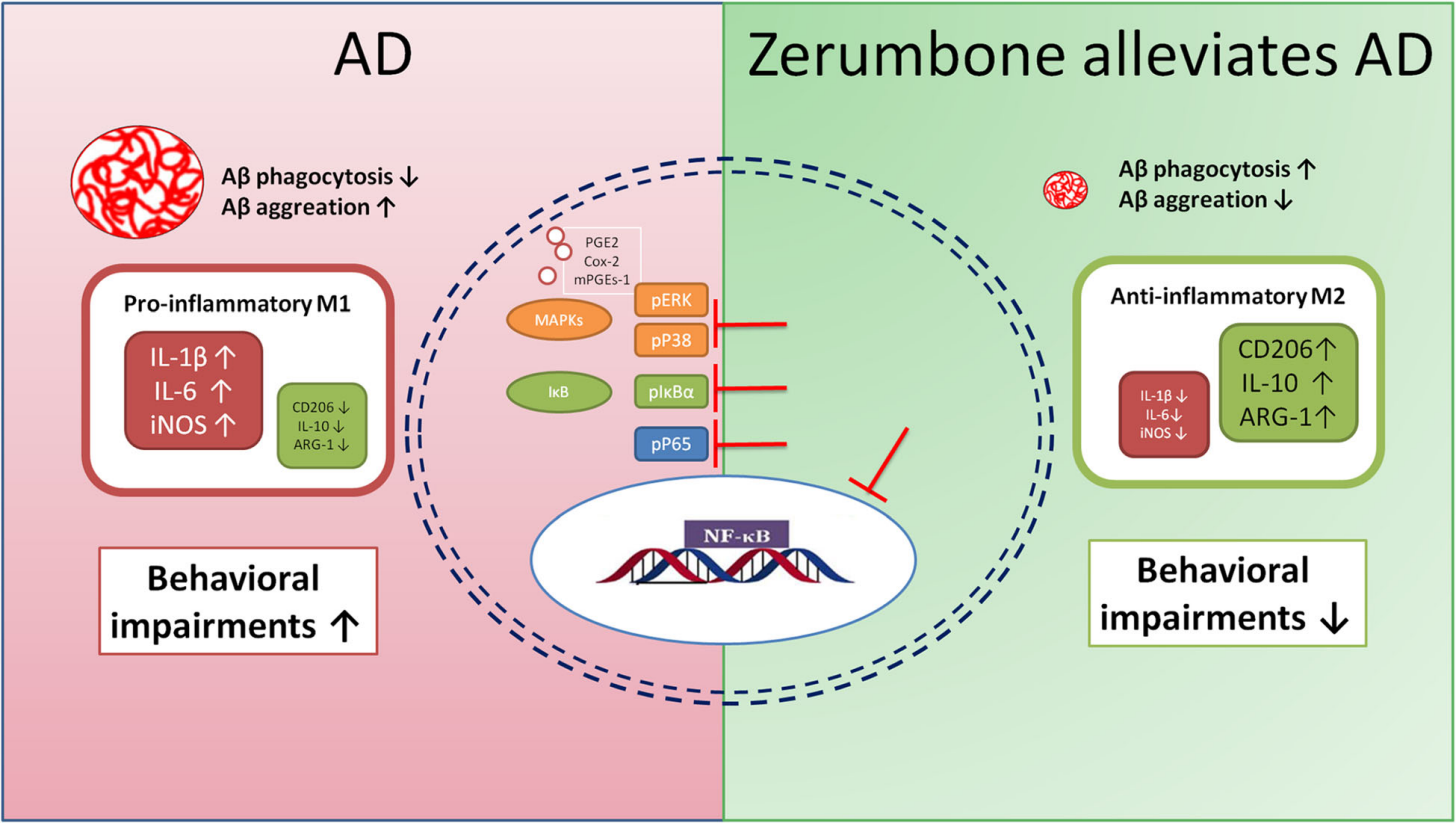
Schematic drawing depicting the suppression of MAPK signaling in microglia to alleviate AD by zerumbone. In the Alzheimer’s disease (AD) brain, β-amyloid (Aβ) induces the release of interleukin-1β (IL-1β), interleukin-6 (IL-6), and inducible nitric oxide synthase (iNOS) and activates inflammatory microglia, suppressing Aβ phagocytosis and promoting behavioral impairments (left, red). In this study, zerumbone inhibited the production of prostaglandin E2 (PGE2), cyclooxygenase-2 (Cox-2), and microsomal prostaglandin E synthase-1 (mPGEs-1). It also increased the proportion of anti-inflammatory microglia and ameliorated behavioral impairments and neuropathological changes in transgenic APP/PS1 mice by suppressing mitogen-activated protein kinase (MAPK) signaling (green). The inhibition of p-extracellular signal-related kinase (p-ERK), p-p38, and nuclear factor-kappa B (NF-κB) was a critical mechanism underlying the neuroprotective effect of zerumbone in microglia (right)

In conclusion, we demonstrated that zerumbone exhibited neuroprotective effects in the transgenic APP/PS1 AD mouse model, ameliorating neuroinflammation and cerebral amyloidosis, thus restoring non-cognitive and cognitive behavioral impairments. This neuroprotective effect of zerumbone may result from its anti-neuroinflammatory activity. Notably, zerumbone switched classically activated pro-inflammatory microglia into anti-inflammatory microglia through regulating the ERK/p38 MAPK signaling pathway, thus enhancing the phagocytosis of Aβ. Taken together, these results suggest that zerumbone may be a promising therapeutic agent for the treatment of human AD (Fig. 7).

## Data Availability

The datasets generated and/or analyzed during the current study are available from the corresponding author on reasonable request.

## Abbreviations

AD: Alzheimer’s disease
APP: Amyloid precursor protein
ARG-1: Arginine-1
Aβ: β-amyloid
BSA: Bovine serum albumin
CCK-8: Cell counting kit-8
CMC: Carboxymethylcellulose
CNS: Central nervous system
Cox-2: Cyclooxygenase-2
ELISA: Enzyme-linked immunosorbent assay
ERK: Extracellular signal-regulated kinase
GFAP: Glial fibrillary acidic protein
HBSS: Hank’s balanced salt solution
Iba-1: Ionized calcium-binding adaptor molecule-1
IL: Interleukin
iNOS: Inducible nitric oxide synthase
JNK: C-Jun N-terminal kinase
LPS: Lipopolysaccharide
MAPK: Mitogen-activated protein kinase
mPGES-1: Microsomal prostaglandin E synthase-1
MWM: Morris water maze
NF-κB: Nuclear factor-kappa B
NO: Nitric oxide
PGE2: Prostaglandin E2
PS1: Presenilin 1
RI: Recognition index
SEM: Standard error of the mean
TNF-α: Tumor necrosis factor-α
